# A TriNetX Analysis of Hypertrophic Scarring Disorders, Genitourinary Strictures, and Urethroplasty Failure

**DOI:** 10.3390/jcm14020302

**Published:** 2025-01-07

**Authors:** Zachary J. Prebay, John Wahlstedt, Afzal Shakir, Eric Wahlstedt, Paul H. Chung, Mihir S. Shah

**Affiliations:** 1 Thomas Jefferson SKMC, 1025 Walnut Street Suite 1100, Philadelphia, PA 19107, USA; john.wahlstedt@students.jefferson.edu (J.W.); afzal.shakir@jefferson.edu (A.S.); paul.chung@jefferson.edu (P.H.C.); mihir.shah@jefferson.edu (M.S.S.); 2 UK College of Medicine, Lexington, KY 40506, USA; erwa234@uky.edu

**Keywords:** urethral stricture, bladder neck contracture, keloid

## Abstract

**Background:** Urethral strictures and bladder neck contractures (BNCs) can be significantly morbid for patients and may require intervention for effective urinary drainage. We hypothesized patients with abnormal scarring disorders, such as keloids or hypertrophic scars, are at elevated risks of urethroplasty failure as well as postprocedural urethral strictures and BNCs. **Methods:** We queried the TriNetX database to determine the risk of urethroplasty failure for patients with abnormal scarring disorders compared to controls. We also investigated the risk of developing urethral strictures and BNCs for patients undergoing various endourology procedures. Results are reported in terms of risk ratio (RR) with 95% confidence interval (CI). Statistical significance was considered when the CI did not include 1.0. Propensity score matching was performed to limit confounding. Notably, TriNetX rounds values < 10 to 10 for patient anonymity (denoted by *). **Results:** Urethroplasty patients with scarring pathology needed a second procedure more than twice as often (36.2% vs. <17.2%*, RR = 2.1, 95%CI 1.1–4.1). Following cystoscopy, there was no difference in urethral stricture rates for patients with scarring disorders (2.7% vs. 2.6%, RR = 1.1, 95%CI 0.85–1.3). These patients also showed similar rates of BNCs (7.5% vs. 5.3%, RR = 1.4, 95%CI 0.84–2.3) and urethral strictures (5.9% vs. 5.3%, RR = 1.1, 95%CI 0.68–1.8) after transurethral bladder outlet procedures. **Conclusions:** Patients with scarring disorders showed much higher urethroplasty failure rates. They experienced similar rates of urethral strictures and BNC formation after endoscopic procedures. These novel findings underscore the importance of recognizing abnormal scarring conditions during preoperative assessments, guiding clinicians in counseling patients and tailoring operative interventions.

## 1. Introduction

Genitourinary strictures, the scarring of urothelial lumens, can introduce significant morbidity, leading to a negative impact on a patient’s quality of life. Strictures can arise from various causes, including trauma, infection, and unfortunately, iatrogenic factors, with the latter being the most prevalent etiology [1]. Scarring in the urinary tract may require procedures to bypass or remove the fibrotic tissue to ensure effective urinary drainage. The ability of urologists to prevent stricture formation by identifying high-risk patients could have important clinical significance.

There are patient factors that may predispose stricture formation, but these factors are not all identified or well understood. Further research into stricture formation would be of benefit, as presently it remains lacking. In particular, there is no research regarding abnormal wound-healing conditions, such as keloid scars and hypertrophic scars, and the urinary tract. Keloid scars form due to increased collagen and extracellular matrix formation and a corresponding decrease in the degradation of these proteins [2]. Most keloids are located around anterior chest, upper back, shoulders and earlobes [3]. Another variant in wound healing is the hypertrophic scar. While excess connective tissue in keloid scarring extends beyond original wound parameters, hypertrophic scarring is contained within the original wound [4]. We do not know whether scarring disorders extend to the urothelium and sought to evaluate this relationship for the first time.

To better understand whether patients with scarring pathology are at increased risk of genitourinary scarring, we utilized the TriNetX database to evaluate whether patients with scarring pathology have the same success following urethroplasty. Additionally, we evaluated the prevalence of urethral strictures and bladder neck contractures in patients following transurethral procedures. We hypothesize that patients with increased risk of keloid and hypertrophic scarring will see lower success following urethroplasty. We also hypothesize patients will have a higher incidence of developing strictures after undergoing endourology procedures. If true, these data may help providers and patients when determining procedural plans to attempt to minimize interventions, thus reducing stricture formation.

## 2. Materials and Methods

We accessed the TriNetX Research Network, which is a collaborative research enterprise containing real-time data from the electronic health records of over 100 million patients located in 89 healthcare organizations worldwide at the time of analysis. The database continually updates but only reports data coded into a patient’s EHR from up to 20 years prior to the date of analysis (2004–2024), excluding those undergoing the index event prior to this date. The dataset includes data on demographics, medical diagnoses, procedures, lab values, and medications. Due to the de-identified nature of this dataset, our study was considered exempt from Institutional Review Board approval (for more information, please visit https://trinetx.com/real-world-resources/case-studies-publications/trinetx-publication-guidelines/, accessed on 18 December 2024). Documentation in TriNetX was reliant on a combination of International Classification of Diseases (ICD) and Current Procedural Terminology (CPT) coding. TriNetX maps ICD-9 and ICD-10 codes to the ICD-10-Clinical-Modification extension.

Cohorts were established based on the presence of hypertrophic scarring disorder (ICD L91.0) or not. We then compared scar formation after two separate types of urologic procedures.

Our primary aim was to determine whether urethroplasty failure differed between abnormal scar forming and control groups. We defined failure as need for a second intervention, including dilation (53600, 53601, 53605), urethrotomy (52275, 52276), and repeat urethroplasty (53400, 53410), following index urethroplasty (CPT 53410) (Table 1), which was consistent with the previous literature [5]. We did not further investigate urethroplasty success based on the location of the stricture or type of repair given the overall low sample sizes.

Our secondary aim was to determine urethral stricture (ICD N35 & ICD N99.1) and bladder neck contracture (“BNC”, ICD N32) formation after index procedure between abnormal scarring and control groups. We first investigated urethral stricture formation following cystoscopy (CPT 52000), as this may be thought of as the most fundamental of urethral interventions. This includes all patients in the TriNetX database who had this specific CPT code in the chart. We also investigated urethral stricture and BNC formation following various transurethral bladder outlet procedures (Transurethral Resection of Prostate 52601, Transurethral Incision of Prostate 52450, Laser Enucleation of Prostate 52649, Laser Vaporization of Prostate 52648, Table 1). Patients were excluded if they carried the diagnosis of investigation prior to index procedure. We did not isolate stricture formation to a single procedure or exclude whether patients had other procedures performed at the same time or following an index procedure. TriNetX does not distinguish whether cystoscopy was performed in the office or hospital setting and similarly does not distinguish between flexible and rigid cystoscopy, as it solely is defined by CPT value. Additionally, we excluded patients with a history of prostatectomy (55801, 55810, 55812, 55815, 55821, 55831, 55840, 55842, 55845, 55866) to limit the possible inclusion of vesicourethral anastomotic strictures which use the same ICD code.

To reduce the impact of confounding, we performed propensity score matching (PSM) on our cohorts for all analyses. TriNetX provides a built-in feature for PSM with specified variables of interest, utilizing 1:1 greedy-nearest neighbor matching to generate equal sized cohorts. Clinical variables included in matching were Age at index (years), Diabetes Mellitus (ICD E08-E13), Chronic Ischemic Heart Disease (ICD I25), history of nicotine dependence (ICD Z87.891) and personal history of irradiation (ICD Z92.3), as these were thought to possibly predispose patients to poor wound healing. We also included African-American/Black race as a covariate given the known variance in keloid incidence amongst racial groups [6]. We chose these factors with the understanding that there likely are other risk factors for stricture development not included in our matching but that it would be impossible to account for all possible risk factors and instead focus on some of the most common risk factors. Statistics are reported in terms of risk ratio (RR) with 95% confidence interval (95%CI). The 95%CIs that did not include 1.0 were considered significant. All analyses were performed internally via TriNetX. Of note, for analyses that result in less than or equal to 10 patients, TriNetX rounds the value to 10 as a part of a patient privacy protection mechanism (denoted by *).

## 3. Results

Analysis was conducted in April 2024. Following urethroplasty, patients with scarring pathology needed a second procedure at over double the rate compared to control patients (Table 2 36.2% vs. <17.2%*, RR = 2.1, 95%CI 1.1–4.1). There were 58 patients in each cohort following matching with a mean age of 45.6 and 42.2 years, respectively, and similar rates of covariates. Regarding other risk factors that may increase the risk of urethral stricture, such as lichen sclerosus or prior urethral trauma, less than 10 patients in the hypertrophic scarring group had these conditions, and thus TriNetX rounds the value for patient anonymity. We are therefore unable to draw better conclusions on the role these factors may play and their relationship to hypertrophic scarring disorders.

Following cystoscopy, patients with scarring disorders developed a urethral stricture 2.7% of the time compared to 2.6% in the comparison group (RR = 1.1, 95%CI 0.85–1.3) (Table 3). Patients with scarring disorders were no more likely to develop both BNC (7.5% vs. 5.3%, RR = 1.4, 95%CI 0.84–2.3) and urethral strictures (5.9% vs. 5.3%, RR = 1.1, 95%CI 0.68–1.8) after transurethral procedures. For the cystoscopy analysis, both groups contained 6028 patients after matching. Following matching, the mean age was 58.7 years with similar rates of the covariates used for PSM. For the transurethral bladder outlet procedure analysis, there were 553 patients in each cohort after matching. The mean age for these patients was 69.9 years and, similarly, the covariates were matched after PSM.

## 4. Discussion

Hypertrophic and keloid scars have been extensively studied across various medical specialties [7,8]. The inflammatory processes associated with keloid and hypertrophic scarring are believed to result from a complex interplay of factors, including M2 macrophages, lymphocytes with an increased CD4:CD8 ratio, mast cells, fibroblasts, myofibroblasts, and genetic components [9,10,11,12,13]. Urologic strictures, commonly occurring due to infections or iatrogenic causes, share similar inflammatory and fibrotic mechanisms [14]. However, despite a substantial body of research on treatment methods for urological strictures, the analysis of iatrogenic causes and their relationships with underlying factors, such as scarring disorders, remains poorly understood. Our analysis suggests there is not a significantly elevated risk of urothelial scarring in patients with preexisting keloid and hypertrophic scarring following endourology procedures. However, and most strikingly, patients with scarring pathology had considerably worse outcomes following urethroplasty.

Our primary aim was to compare urethroplasty success rates and the need for additional interventions following urethroplasty, which showed striking disparities between groups. Our group previously found that within TriNetX, 14.3% of patients need a second procedure following urethroplasty [5], which was comparable with previous single-center data [15]. However, when we separated out the patients with scarring disorders, we found that these patients were over twice as likely to need a second procedure, 36.2% of the time compared to <17.2%* in control patients. This finding is important for patient counseling. Surgeons must be aware that a patient with scarring pathology would be expected to fail urethroplasty at a higher rate and perhaps may benefit from alternative intervention. Additionally, limiting events that may result in stricture formation in the first place should be taken into consideration as prevention is better than cure. What is unknown is whether patients with scarring disorders undergoing urethroplasty may experience different outcomes whether they undergo primary anastomotic repair or substitution repair including grafting or flaps. This topic may be worthy of further investigation.

For control patients, our findings corroborate urethral stricture and BNC formation previously published in the literature. In a 2010 meta-analysis by Ahyai et al., they report urethral stricture rates from 1.9 to 6.3% and BNC rates from 0.5 to 5.0% based on various transurethral bladder outlet procedures [16]. Our findings within the TriNetX database are similar, as 2.6% of patients post-cystoscopy and 5.3% of control patients post-transurethral intervention experienced urethral strictures. We observed a slightly elevated incidence of BNC among patients with scarring disorders undergoing transurethral procedures, from 5.3% in control patients to 7.5% in our scarring group, but again not to the level of statistical significance. We did not evaluate the impact of intraoperative risk factors, such as gland volume or resection size, which have been studied previously but would not be possible to capture in the TriNetX database [17,18]. However, to reduce confounding, we did include multiple patient factors in our PSM, which were similar to those reported in these previous studies. Perhaps it would be possible to identify alterations in operative techniques that lead to reduced stricture and BNC formation and would be of benefit in this specific population, even if the technique were to come at the expense of efficacy or not be generalizable to all patients.

As a retrospective study, we are unable to conclude any causative effect but do conjecture some inferences from our data and generate hypotheses. In a sense, cystoscopy can be thought of as a “fundamental or basic” procedure compared to more extensive transurethral resection that likely includes the use of a resectoscope, which is a larger instrument typically. Overall, there was a higher rate of urethral stricture formation following transurethral resection procedures compared to cystoscopy. This does suggest the larger instruments that are typically used in outlet procedures do result in higher stricture rates, which does empirically make sense to urologists who may notice more urethral trauma with the insertion of larger instruments such as resectoscopes compared to cystoscopes. However, we did not account for the number of cystoscopies or procedures patients may have underwent. The patients with scarring pathology did have a numerically higher rate of urethral stricture compared to control patients following transurethral intervention but not to the level of statistical significance. These patients did also have slightly increased rates of BNC following transurethral procedures. This implies that the passage of an instrument alone, and the disruption of urothelium that occurs as a result, can lead to stricture formation, and we suggest this result is amplified by patients with scarring disorders. Further, we suggest that the act of cutting urothelium (whether via loop, knife, or laser) results in an even more pronounced difference in scarring postoperatively when comparing those with scarring disorders and control patients.

While it is perhaps unsurprising that patients with abnormal scarring elsewhere in the body also experience higher rates of scarring in the urinary tract, there has been little attention paid to these patients in urology previously. One such study by Aydemir et al. had previously highlighted a connection between increased urethral stricture length in patients with poorly healed median sternotomy incision scars [19]. Their study was limited to 368 patients undergoing internal urethrotomy and was conducted within a single institution in Turkey. This group appears to have identified a similar cohort with abnormal scar formation. We more specifically define our study population by utilizing ICD diagnosis to identify our cohort. Additionally, we investigate various urothelial scarring across multiple endourologic interventions as well as success following urethroplasty, generating more robust data.

This study is not without limitations, primarily those related to performing a retrospective analysis using a registry database outside the investigators control. We acknowledge that our findings should be considered corollary and hypothesis generating but that no causation can be ascribed. Additionally, although we performed PSM using common risk factors for stricture formation, there are likely additional risk factors that are worthy of consideration that either could not or were not included in our PSM. This study relies upon the assumption that patients are accurately being diagnosed with keloid scars in the TriNetX database. In prospective study, patients could be asked about abnormal scarring at preoperative appointments. TriNetX lacks specific patient and operative descriptors, confining analyses to generic ICD and CPT codes, including factors such as whether patients required a catheter before or after a procedure, which may be an independent risk factor for strictures. Furthermore, the rationale behind the procedure and complication selection using these codes cannot be determined with this database. Additionally, we did not restrict this analysis to patients undergoing a single procedure, which may have falsely elevated our results. For example, someone undergoing multiple cystoscopy procedures may be more likely to develop a stricture compared to someone only having one cystoscopy. We also were unable to identify the instruments used, and perhaps certain brands of devices or caliber of instruments would be more or less likely to result in urothelial disruption. The absence of a published list of healthcare organizations comprising the TriNetX network limited our ability to gauge factors like fellowship-trained providers in the urethroplasty success analysis. We also were limited by a relatively low cohort size for patients with scarring pathology undergoing urethroplasty. TriNetX primarily draws data from healthcare organizations, which largely insure patients within large employer systems. Discrepancies in coverage, particularly regarding out-of-pocket expenses and out-of-network costs, might have influenced patients’ healthcare-seeking behavior and the timing of urological care, thus limiting the conclusiveness and applicability of the data beyond general trends. We also may miss patients who split their care between different institutions if one is in TriNetX and another not. Although this paper strives to draw attention to elevated stricture rates post-urological procedures, caution should be exercised in generalizing these findings to make specific conclusions about stricture incidence.

Highlighting this study’s strengths, to the best of our knowledge, this is the first in-depth look at the relationship of abnormal scarring pathology and the urinary tract. We analyzed a substantial patient population from multiple healthcare institutions, a departure from smaller, single- or few-institution studies, which are more common in reconstructive urology. Notably, the 2023 AUA guidelines omit any mention of abnormal scarring, emphasizing the need for additional research [20]. This work is of interest to both patients and providers, as it has the potential to improve counseling, reduce complications and enhance post-surgical patient care. Future investigations focused on scarring complications during urological procedures could lead to more effective clinical strategies, ultimately benefiting patient outcomes and cost-effectiveness. Preoperatively, asking patients about their scarring history could assist with setting postoperative expectations. Intraoperatively, identifying techniques to better repair strictures amongst patients with scarring disorders would be of great benefit to this population. Postoperatively, providers could consider resting the urethra for longer or increasing follow-up analysis to assess for stricture occurrence. And finally, the exact pathophysiology of how the urethra heals differently in patients with scarring disorders remains unknown. Conditions such as keloid scarring and hypertrophic scarring result from disorders related to collagen, which is also involved in stricture formation. Therefore, there is a possible relationship between these skin conditions and the urethra. Further basic science research into the exact mechanism by which the urethra would scar differently in patients with abnormal scarring would help us understand this phenomenon further.

## 5. Conclusions

This retrospective, multi-institutional database study suggests a strong relationship between abnormal scarring disorders and urethroplasty failure. On the other hand, there was no statistically increased risk of urethral stricture or BNC following endourology procedures. Despite data limitations, these findings underscore the need for a heightened awareness of scarring conditions in preoperative assessments and warrant further investigation. Recognizing these associations could lead to improved clinical strategies, enhancing patient outcomes, and reducing healthcare morbidity in urology. Further research is essential to validate these findings and measure impact on clinical practice.

## Figures and Tables

**Table 1:** International Classification of Disease and Current Procedural Terminology Codes.

**International Classification of Disease (ICD) Code**	**Outcome**
E08-E13	Diabetes Mellitus
I25	Chronic Ischemic Heart Disease
L91.0	Hypertrophic scar
N32.0	Bladder neck obstruction
N35	Urethral stricture
N99.1	Postprocedural urethral stricture
Z92.3	Personal history of irradiation
Z87.891	History of nicotine dependence
**Current Procedural Terminology (CPT) Code**	**Outcome**
52000	Cystourethroscopy
52275	Cystourethroscopy, with internal urethrotomy; male
52276	Cystourethroscopy with direct vision internal urethrotomy
52450	Transurethral incision of prostate
52601	Transurethral electrosurgical resection of prostate, including control of postoperative bleeding, complete (vasectomy, meatotomy, cystourethroscopy, urethral calibration and/or dilation, and internal urethrotomy are included)
52648	Laser vaporization of prostate, including control of postoperative bleeding, complete (vasectomy, meatotomy, cystourethroscopy, urethral calibration and/or dilation, internal urethrotomy and transurethral resection of prostate are included if performed)
52649	Laser enucleation of the prostate with morcellation, including control of postoperative bleeding, complete (vasectomy, meatotomy, cystourethroscopy, urethral calibration and/or dilation, internal urethrotomy and transurethral resection of prostate are included if performed)
53400	Urethroplasty; first stage, for fistula, diverticulum, or stricture
53410	Urethroplasty, one-stage reconstruction of male anterior urethra
53600	Dilation of urethral stricture by passage of sound or urethral dilator, male; initial
53601	Dilation of urethral stricture by passage of sound or urethral dilator, male; subsequent
53605	Dilation of urethral stricture or vesical neck by passage of sound or urethral dilator, male, general or conduction (spinal) anesthesia
55801	Prostatectomy, perineal, subtotal (including control of postoperative bleeding, vasectomy, meatotomy, urethral calibration and/or dilation, and internal urethrotomy)
55810	Prostatectomy, perineal radical
55812	Prostatectomy, perineal radical; with lymph node biopsy(s) (limited pelvic lymphadenectomy)
55815	Prostatectomy, perineal radical; with bilateral pelvic lymphadenectomy, including external iliac, hypogastric and obturator nodes
55821	Prostatectomy (including control of postoperative bleeding, vasectomy, meatotomy, urethral calibration and/or dilation, and internal urethrotomy); suprapubic, subtotal, 1 or 2 stages
55831	Prostatectomy (including control of postoperative bleeding, vasectomy, meatotomy, urethral calibration and/or dilation, and internal urethrotomy); retropubic, subtotal
55840	Prostatectomy, retropubic radical, with or without nerve sparing
55842	Prostatectomy, retropubic radical, with or without nerve sparing; with lymph node biopsy(s) (limited pelvic lymphadenectomy)
55845	Prostatectomy, retropubic radical, with or without nerve sparing; with bilateral pelvic lymphadenectomy, including external iliac, hypogastric, and obturator nodes
55866	Laparoscopy, surgical prostatectomy, retropubic radical, including nerve sparing, includes robotic assistance, when performed

**Table 2:** Urethroplasty success based on scarring pathology.

	N After PSM	Second Procedure	RR 95% CI
Scarring Disorder Urethroplasty	58	36.2%	2.1
Urethroplasty	58	<17.2% *	1.1–4.1

N—number, RR—risk ratio, CI—confidence interval. Of note, for analyses that result in less than or equal to 10 patients, TriNetX rounds the value to 10 as a part of a patient privacy protection mechanism (denoted by *).

**Table 3:** Stricture formation following endourologic procedures based on scarring pathology.

Scarring Disorder	Procedure	N After PSM	Urethral Stricture	RR 95% CI	BNC	RR 95% CI
Yes No	Cystoscopy	6028 6028	2.7% 2.6%	1.1 0.85–1.3	X	X
Yes No	Transurethral Intervention	553 553	5.9% 5.3%	1.1 0.69–1.8	7.5% 5.3%	1.4 0.84–2.3

N—number, PSM—propensity score matching, RR—risk ratio, CI—confidence interval, BNC—bladder neck contracture.

## Data Availability

The data used in this article is proprietary to TriNetX and available via institutional license.
